# Unilateral Hyperlucent Lung in Adulthood: A Case Report of Swyer-James-Macleod Syndrome

**DOI:** 10.7759/cureus.54107

**Published:** 2024-02-13

**Authors:** Praveen K Sharma, Sakthi Ganesh Subramonian, Karpagam R K, Sanjaykanth B, Yashaswinii Polaka

**Affiliations:** 1 Department of Radiology, Saveetha Medical College and Hospital, Saveetha Institute of Medical and Technical Sciences (SIMATS) Deemed University, Chennai, IND

**Keywords:** hyperlucent, lung, computed tomography, x-ray, middle aged

## Abstract

Swyer-James-Macleod syndrome (SJMS) or Bret syndrome presents as unilateral hyperlucent lung, an uncommon pulmonary condition. The accurate diagnosis of SJMS requires high-resolution CT (HRCT), as conventional chest radiographs may underestimate this condition. We present a case of SJMS in a 54-year-old male who was managed with bronchodilators and intravenous antibiotics. This case report underscores the rarity of SJMS in adulthood, with only a limited number of cases reported globally so far. Comprehensive use of HRCT is crucial for precise diagnosis, and early intervention with appropriate medical management is imperative for favorable outcomes.

## Introduction

In 1953, Paul Robert Swyer, a Canadian pediatrician, and George C. W James, a Canadian radiologist, first reported the condition that eventually came to be known as Swyer-James-Macleod syndrome (SJMS). William Mathieson MacLeod, an English pulmonologist, subsequently provided extensive details about the unilateral hyperlucent lung [1,2]. SJMS, or unilateral hyperlucent lung, is a rare pulmonary condition secondary to post-infectious bronchiolitis obliterans in childhood [3], arising as a chronic consequence of infantile and pediatric bronchiolitis, particularly following an adenoviral infection. This illness can occasionally present with mild symptoms or be asymptomatic, and a diagnosis may be delayed until patients attain maturity. SJMS is characterized by pulmonary arterial hypoplasia and agenesis. It results in pulmonary parenchyma hypoperfusion and is associated with a translucent or hyperlucent unilateral lung [4].

## Case presentation

A 54-year-old male presented to the emergency room with a one-month history of dry cough and dyspnea without expectoration. No fever or hemoptysis was reported. His medical history revealed recurrent respiratory infections in childhood and early adulthood, but no bronchial asthma, systemic hypertension, diabetes mellitus, or tuberculosis. He had been a chronic smoker for 20 years, with no significant family medical history.

Physical examination showed a pulse rate of 86 bpm, blood pressure at 130/80 mmHg, respiratory rate of 28/min, temperature at 98.2 °F, and SPO_2_ of 98%. Wheezing was noted, but no cyanosis or clubbing. Auscultation revealed crackles in the left hemithorax. Laboratory investigations were significant for an elevated white blood cell count of 11900/mL, indicating a possible infection. Pulmonary function tests showed a reduced forced expiratory volume in one second (FEV1) and forced vital capacity (FVC), with an FEV1/FVC ratio within normal limits, suggesting a restrictive lung disease pattern. Arterial blood gas analysis showed a mild hypoxemia with a PaO_2_ of 75 mmHg.

**Figure 1:**
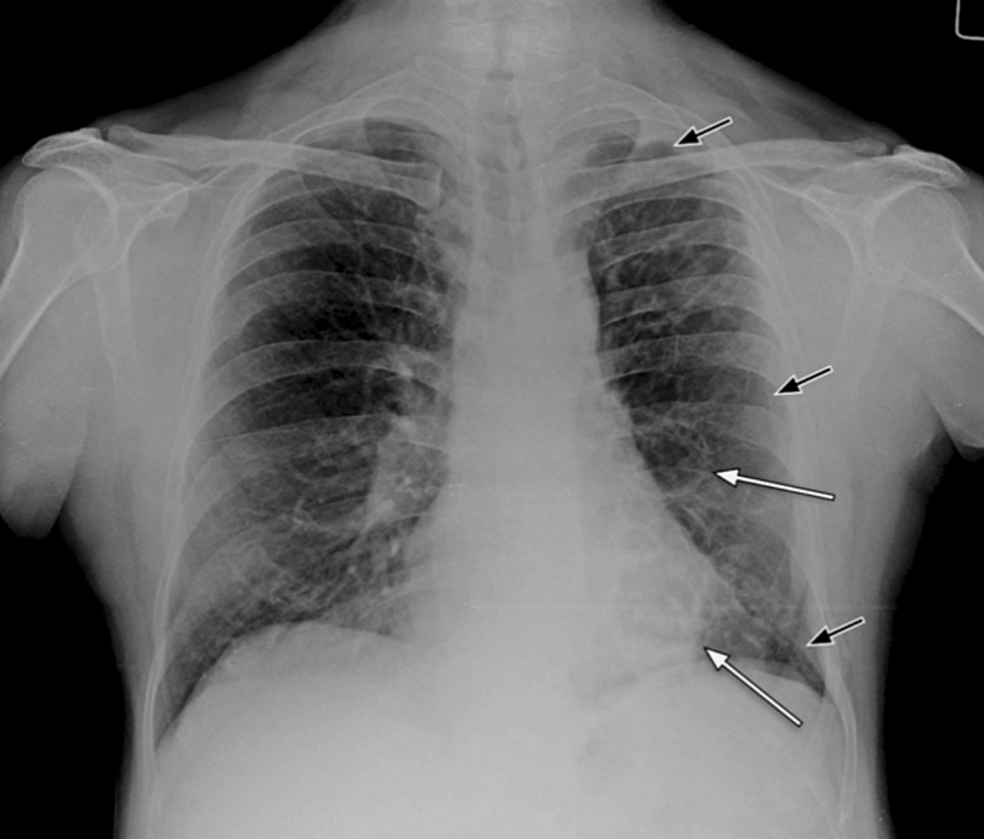
Initial frontal chest radiograph The image shows unilateral small left lung (short black arrows) and cystic bronchiectasis in the left mid and lower zone lung fields and a few air-fluid levels in the cystic changes (long white arrows)

**Figure 2:**
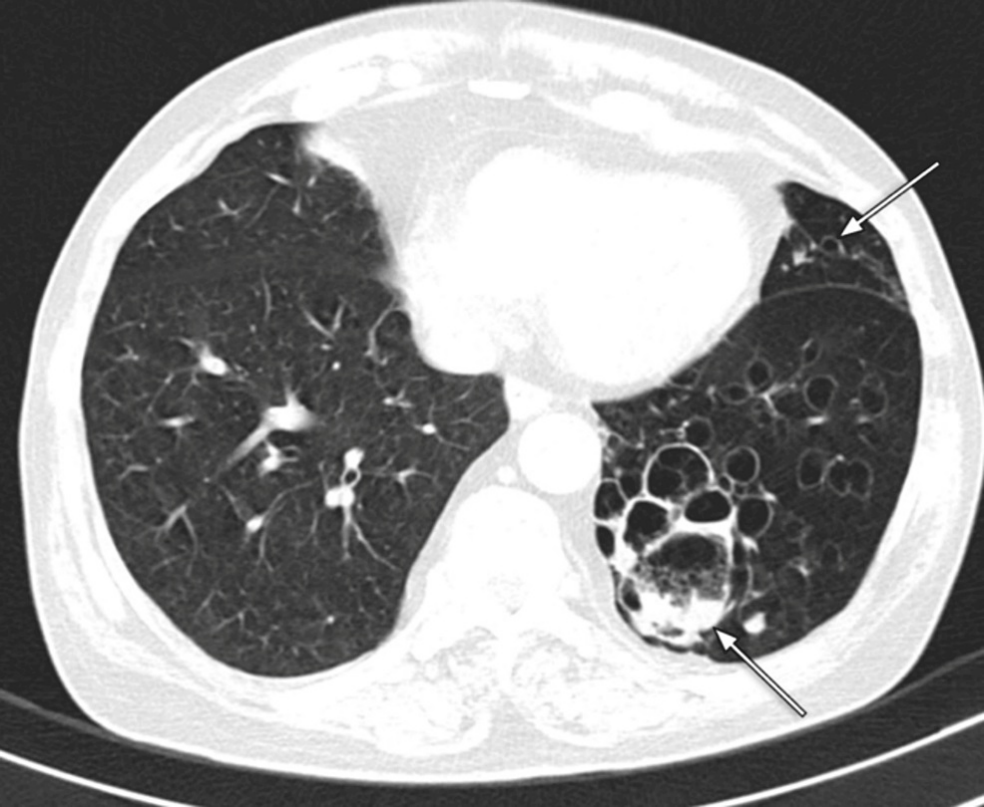
HRCT chest (axial section in lung window) The image shows cystic bronchiectasis and bronchiolectasis in the left lung and a few air-fluid levels in the cystic changes (long white arrows) HRCT: high-resolution computed tomography

**Figure 3:**
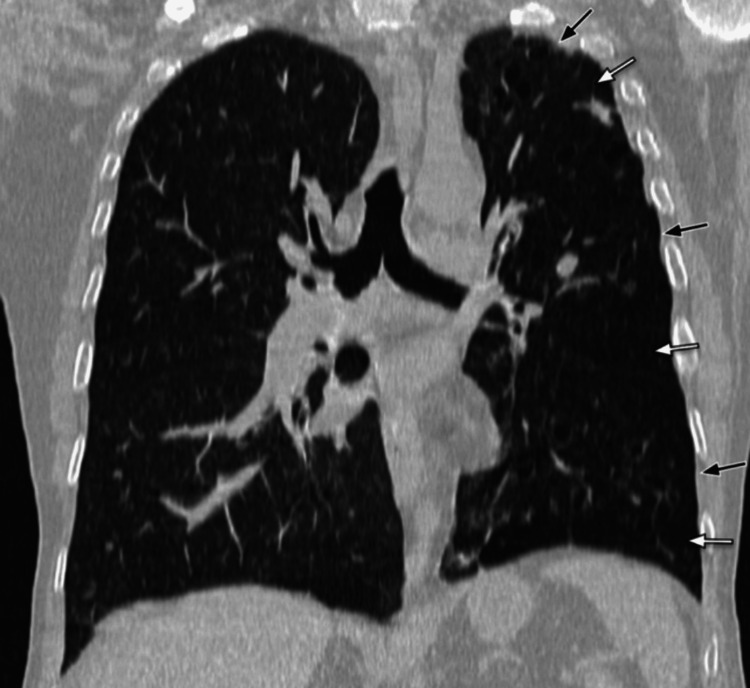
HRCT chest with maximum intensity projection (coronal section) The image shows unilateral small lung (short black arrows) and unilateral diffuse oligemia in the left lung (short white arrows) HRCT: high-resolution computed tomography

**Figure 4:**
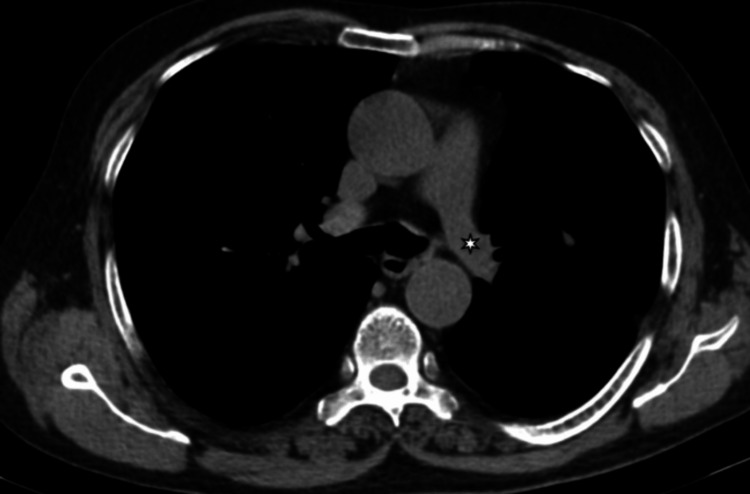
HRCT chest (axial section in the mediastinal window) The image shows the left pulmonary artery small in caliber/hypoplastic (small white asterisk) HRCT: high-resolution computed tomography

**Figure 5:**
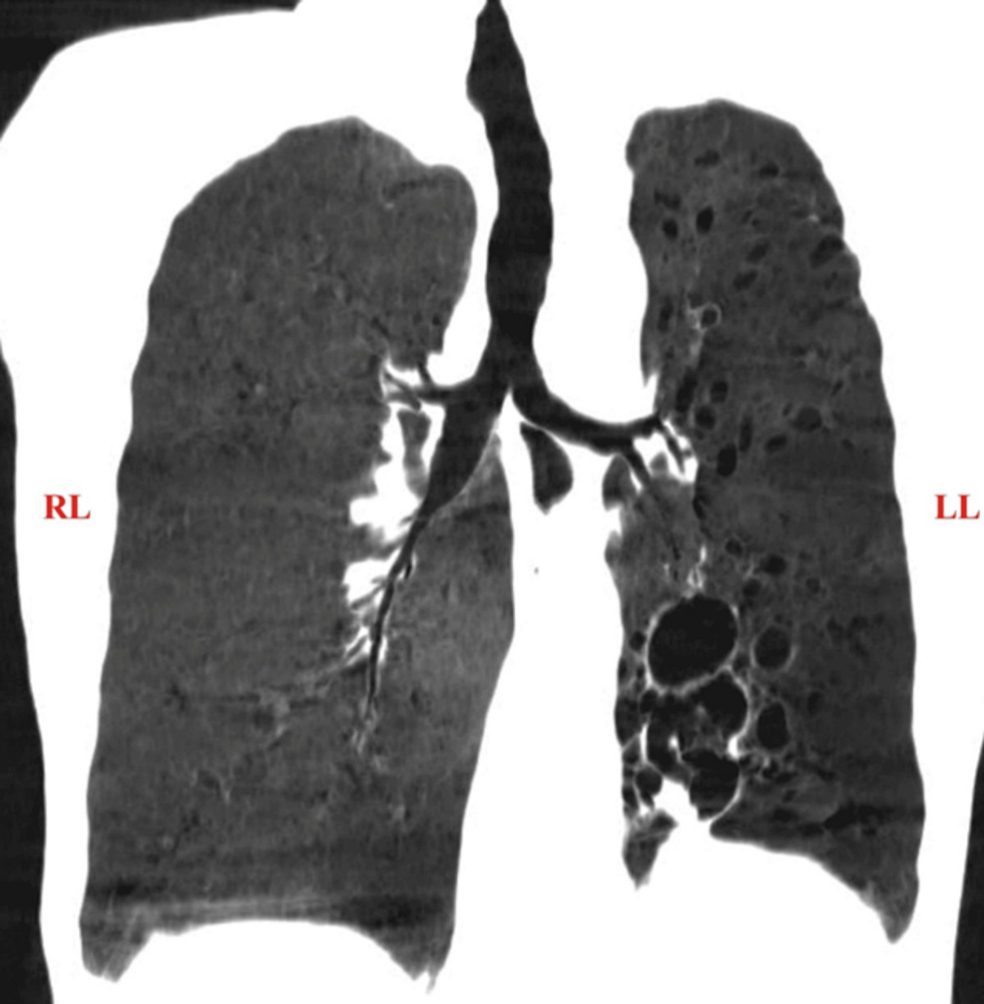
HRCT chest with minimum intensity projection (coronal section) The image shows cystic bronchiectasis and bronchiolectasis in the left lung HRCT: high-resolution computed tomography; RL: right lung; LL: left lung

**Figure 6:**
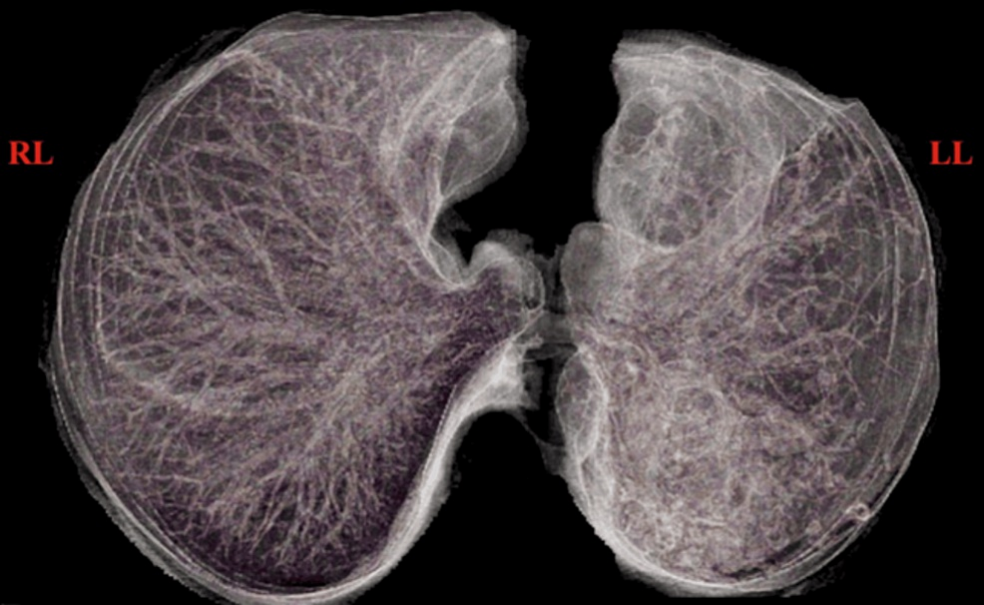
HRCT chest with (3D) volume-rendering The image shows unilateral diffuse oligemia in the left lung HRCT: high-resolution computed tomography; RL: right lung; LL: left lung

An initial chest radiograph (Figure 1) indicated a unilateral small left lung, with cystic bronchiectasis in the left mid- and lower-zone lung fields, and some cysts showing an air-fluid level. Subsequent high-resolution CT (HRCT) of the chest further detailed a unilateral small left lung with diffuse oligemia, extensive cystic bronchiectasis, and bronchiolectasis throughout all segments of the left lung. A few cysts exhibited air-fluid levels. The left pulmonary artery was observed to have a maximum diameter of 12 mm, suggesting hypoplasia (Figures 2-6).

Based on these findings, a diagnosis of SJMS was made. The syndrome is characterized by post-infectious bronchiolitis obliterans leading to unilateral lung hyperlucency due to air trapping, reduced vascularity, and a small pulmonary artery on the affected side. Following the diagnosis, the patient's treatment plan was meticulously structured to tackle both the immediate symptoms and the underlying condition, while also focusing on long-term respiratory health improvement. Initially, bronchodilators were administered to alleviate the dyspnea and wheezing, with a specific regimen of a long-acting beta-agonist (LABA) combined with an inhaled corticosteroid (ICS) to reduce inflammation within the bronchial passages. Intravenous antibiotics were prescribed, targeting the suspected bronchial infection, with a two-week course of a broad-spectrum antibiotic that was carefully selected based on the patient's past medical history and current presentation. Concurrently, a targeted pulmonary rehabilitation program was introduced, starting with gentle, supervised exercise sessions that gradually increased in intensity, focusing on enhancing lung capacity and strengthening the respiratory muscles. Nutritional counseling was incorporated to support overall health and facilitate the recovery process.

As part of the comprehensive care approach, the patient was enrolled in a smoking cessation program, which included behavioral therapy and pharmacotherapy with nicotine replacement products to address nicotine dependence. This multidisciplinary treatment strategy was aimed not only at managing the immediate symptoms and complications of SJMS but also at significantly improving the patient's quality of life and long-term health outcomes. Regular follow-up appointments were scheduled to monitor the patient's progress, adjust the treatment plan as necessary, and ensure that the patient remained engaged and committed to the smoking cessation and pulmonary rehabilitation programs.

## Discussion

Bronchiolitis with small airway obliteration and emphysema leads to alveolar destruction and dilated lung parenchyma. Peripheral pulmonary vascularization is decreased due to inflammation-bronchiolitis obliterans leading to atelectasis or scarring, resulting in bronchiectasis. The original viral infection harms the bronchioles and bronchi [5]. It is a sequel to pediatric pneumonitis and viral bronchiolitis. The causative organisms include Paramyxovirus morbillivirus, Bordetella pertussis, and Mycobacterium tuberculosis. Mycoplasma pneumoniae, influenza A, measles, and adenovirus types 3, 7, and 21 are also associated with this condition [6].

SJMS or Bret syndrome is usually asymptomatic. The typical clinical manifestations of SJMS in childhood include dyspnea, productive cough, hemoptysis, decreased exercise tolerance, and recurrent pulmonary infections [7]. Compared to a previous study where dyspnea was not a prominent feature, the most common complaint in one study was dyspnea on exertion [8]. The typical clinical manifestations in adulthood include chest pain, wheezing, and hemoptysis. Other manifestations are bronchiectasis, bronchial hyperresponsiveness, recurrent bronchitis, and pneumonia [9]. The complications include lung abscesses, spontaneous pneumothorax, and recurring infection in bronchiectasis [10,11].

A small hilar shadow, displacement of the mediastinum to the affected side, and a unilateral hyperlucent lung with diminished broncho-vascular markings are the hallmarks of chest radiography in this patient population [12]. HRCT chest is characterized by pulmonary artery hypoplasia or agenesis. It leads to pulmonary parenchyma hypoperfusion or diminished vascularity. It results in unilateral hyperlucent lung disease with diffuse oligemia. Bronchiectasis, bronchiolectasis, atelectasis, and scarring may also be present [13]. CT pulmonary angiography is not an essential criterion for entity diagnosis. However, it can reveal pulmonary hypoplasia or agenesis.

The differential diagnosis for unilateral lung hyperlucency involves various conditions with similar radiographic manifestations, and SJMS emerges as a key contender, characterized by post-infectious bronchiolitis obliterans causing obstructive defects in small airways, leading to air trapping and unilateral hyperlucent lung appearance on imaging. Bronchiolitis obliterans, another obstructive lung disease, can also lead to hyperlucent lung areas due to small airway obliteration. Bronchiectasis, known for lung opacity from chronic inflammation, can result in hyperlucency with associated air trapping or lung parenchyma destruction. Congenital pulmonary airway malformation (CPAM) in infancy causes localized lung overinflation and hyperlucency. Pulmonary artery hypoplasia or agenesis reduces blood flow, diminishing lung vascularity and causing hyperlucent lung appearance. Foreign body aspiration in children can lead to air trapping and unilateral hyperlucency. Compensatory overinflation post-surgical lung removal can also cause hyperinflation. Diagnosis requires consideration of clinical history, symptoms, and imaging findings. In the described scenario, a history of viral infection and chronic respiratory symptoms suggested SJMS, supported by diminished bronchovascular markings and pulmonary hypoplasia or agenesis on imaging.

Treatment of SJMS ranges from conservative management to surgical intervention. Traditional therapy includes prompt and adequate antibiotic treatment, as well as influenza and pneumococcal vaccination [14]. Other methods include low-dose inhaled bronchodilators, inhaled corticosteroids, and chest physiotherapy [15]. Long-term oxygen treatment is recommended for respiratory failure and advanced illness. Surgical intervention mainly involves pneumectomy, and some patients undergo lobectomy or segmentectomy [16]. The prognosis is determined by the absence or presence of bronchiectasis. The differential diagnosis includes bronchial asthma, pulmonary artery hypoplasia, congenital lobar emphysema, and pneumothorax [17]. It also includes pulmonary thromboembolism, gastrointestinal herniation, bronchial compression, mastectomy, mediastinal fibrosis, [18], and Poland syndrome [19].

## Conclusions

The present case report highlights the fact that chest radiographs may underestimate the prevalence of the SJMS that presents with unilateral lung hyperlucency. Apart from a chest radiograph, a complementary HRCT study is essential for accurately diagnosing SJMS. Inaccurate diagnoses can lead to inappropriate treatment. Only a few SJMS cases in adulthood have been reported worldwide, which prompted us to prepare this case report to add to the existing body of knowledge on the condition.
